# Unveiling the Myth: Harpy Eagle *Harpia harpyja* Attacks on a Human in the Amazon Forest

**DOI:** 10.1002/ece3.71266

**Published:** 2025-04-24

**Authors:** Loïc Epelboin, Rémi Mutricy, Vincent Pelletier, Alexis Fremery, Maxime Dechelle, Otte Ottema, Sébastien Pfefer, Jenn Sinasac, Sylvain Uriot, Oliver Claessens, Everton B. P. Miranda

**Affiliations:** ^1^ Unité Des Maladies Infectieuses et Tropicales Centre Hospitalier de Cayenne Cayenne France; ^2^ Centre D'investigation Clinique Antilles Guyane, Inserm CIC 1424 Centre Hospitalier de Cayenne Cayenne France; ^3^ Service D'accueil Des Urgences Centre Hospitalier de Cayenne Cayenne France; ^4^ Independant Expert Cayenne France; ^5^ Free‐Lance Photographer Lignieres de Touraine France; ^6^ Stichting Natuurbehoud Suriname (STINASU) Paramaribo Suriname; ^7^ Service D'accueil Des Urgences Centre Hospitalier de Kourou Kourou France; ^8^ Whitehawk—Birding and Conservation Panama City Panama; ^9^ Groupe D'etude et de Protection Des Oiseaux en Guyane (GEPOG) Rémire‐Montjoly France; ^10^ Macroecology Laboratory Graduate School of Life Sciences, Tohoku University Sendai Miyagi Japan

**Keywords:** antipredatory behavior, apex predator, evolutionary dynamics, French Guiana, human–wildlife conflict, primate predation, Taung Child

## Abstract

Predatory interactions between large raptors and primates offer insights into evolutionary dynamics and ecological roles in tropical ecosystems. Harpy Eagles ( *Harpia harpyja* ), known for their size, are generally thought to pose minimal threat to humans, with many studies focusing on diet. However, eagle attacks on humans are exceedingly rare and often anecdotal. Here we show the first scientifically documented case of a Harpy Eagle attacking an adult human in the Amazon rainforest. This finding challenges the assumption that Harpy Eagles do not pose threats to humans outside of nest defense and reveals that, under certain conditions, large raptors may engage in aggressive behavior toward humans. Our results suggest the need to reassess established views on eagle's interactions with humans, contributing to a broader understanding of predator–prey dynamics and highlighting the importance of sociality as an antipredator strategy.

## Graphical abstract

We report here on an unexpected attack by a harpy eagle on a 29‐year‐old woman near a tourist camp in the French Guiana Amazon rainforest.

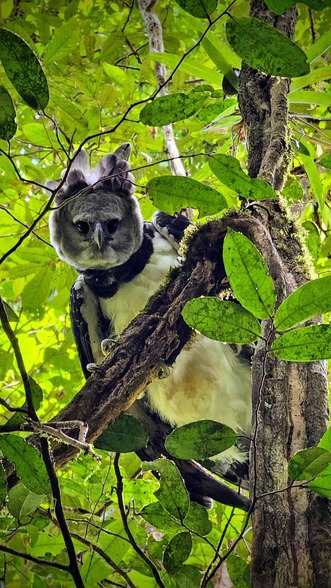

## 1 Introduction

Primate predation holds significant importance in understanding human evolution (Hart and Sussman 2019). The role of raptors as predators for hominids and primates in general offers insights into ecological interactions shaping our ancestral past (Berger and Clarke 1995). Much of the evidence of these interactions comes, unsurprisingly, from Africa. Neotropical predators, on the other hand, exhibit rare instances of human predation, a subject explored by Haddad in his research on various apex predators (jaguars, black caimans, piranhas; Haddad and Fonseca 2011; Haddad and Sazima 2003; Neto, Garrone Neto, and Haddad 2011). Cougars ( *Puma concolor* ) emerge as the most likely human predator across the Neotropics (Mattson et al. 2011). Such examples emphasize the varied but infrequent role of predators in the Neotropics, contrasting with ancient interactions involving raptors elsewhere.

The case of the Taung Child, a young *Australopithecus africanus* possibly preyed upon by an eagle 2.5 million years ago in southern Africa could be considered an idiosyncrasy, since it challenges what is perceived as typical prey size for large eagles (Berger and McGraw 2007). On the other hand, the existence of recently extinct giant raptors such as the Cuban Terrestrial Owl (*Ornimegalonyx oteroi*, up to 9 kg) or the New Zealand’ Haast Eagle (*Hieraaetus moorei*, 9–15 kg) suggests that a few species of large raptors may have been able to overpower infant humans (Suárez 2020; van Heteren et al. 2021). Several fierce raptors coexisted with small humans, anthropoids, and their infants in diverse ecosystems, posing the question of possible interplay between avian predators and humans' evolution (Redfern 2016).

The Harpy Eagle, *Harpia harpyja* , ranks among the heaviest extant raptors globally. Harpy Eagle females exceed males in size, weighing 6–9 kg and 4–5 kg, respectively (Schulenberg 2020). Their prey comprises canopy medium‐sized mammals, encompassing sloths and primates (Garbino et al. 2023; Kaizer et al. 2023). The maximum carrying capacity in flight is uncertain and is supposed to be similar to the eagle's body mass. Thus, a female of 9 kg could carry a prey of 9 kg—whereas evidence still precludes objective testing. Indeed, Harpy Eagles are mighty raptors and documented instances reveal remarkable feats: simultaneous arrival with a spider monkey ( *Ateles chamek* ) and opossum (*Didelphis* spp.) was observed in one nest, while two infant Collared Peccaries ( *Pecari tajacu* ) have been snatched simultaneously, besides records of predation over juvenile peccary that exceeds the eagle's weight (Miranda et al. 2021; Touchton et al. 2002). Harpy Eagles have been observed preying on adult male howler monkeys weighing over 6 kg (Peres 1990; Rettig 1978). Regarding documented attacks on humans, captive‐translocated Harpy Eagles in Panamá displayed aggression toward attendants, prompting the permanent removal of a few individuals from the program (Watson et al. 2016).

There are several descriptions of raptor attacks on humans, outside the context of nest defense or captive birds, but few are scientifically documented. In an interview dedicated to Crowned Eagles ( *Stephanoaetus coronatus* ), Simon Thomsetts describes the following incident in the 1970s in Kenya: “One macabre anecdote is that while investigating an alleged kill of a human infant (4‐year‐old girl) I was brought to the tree where her severed limb was found. The circumstances led to no doubt that the accusation was true for no leopard could have climbed that tree, and nor did the locals know that eagles cached limbs (https://www.africanraptors.org/simon‐thomsett‐on‐the‐african‐crowned‐eagle‐part‐2/). (Thomsett 2015). In 2016, the attack of a Wedge‐Tailed Eagle ( *Aquila audax* ) over a boy during a bird show in Australia was reported by ABC news. In another report, a woman was attacked by a Golden Eagle ( *Aquila chrysaetos* ) in a cage, suffering severe damage to her eyeball (Muller and Kohnen 2005). These cases, summed with the Taung Child, remain as exceedingly rare examples of attacks of raptors on humans.

In this paper, we report on a new instance of Harpy Eagle attack on a human being. This incident sheds light on the broader and significant evolutionary roles of raptors in primate evolution. Our aims are to (a) offer the first documentation of a vicious attack by wild Harpy Eagles on an adult human and (b) contextualize these findings to the previous literature.

## 2 Material and Methods

### 2.1 Study Area Description

The interior of French Guiana is largely uninhabited and covered by tropical forest, with the exception of a few villages scattered along the two rivers on the east and west borders and their major tributaries. The sighting reported in this article took place 30 km from the coast, in an uninhabited area within the Kourou River basin, close to an ecotourism camp. The forest is undisturbed. Here, wildlife–human interactions are limited to occasional sightings by tourists or naturalists.

### 2.2 Literature Review

A study search was made with Google Scholar using *Harpia harpyja* and the following keywords: Harpy Eagle, aria, gavião‐real, harpia, harpie, combined with attack diet (in English and Portuguese). This allowed us to access published and unpublished studies in English, French, Portuguese, and Spanish.

## 3 Case Report

**FIGURE 1:**
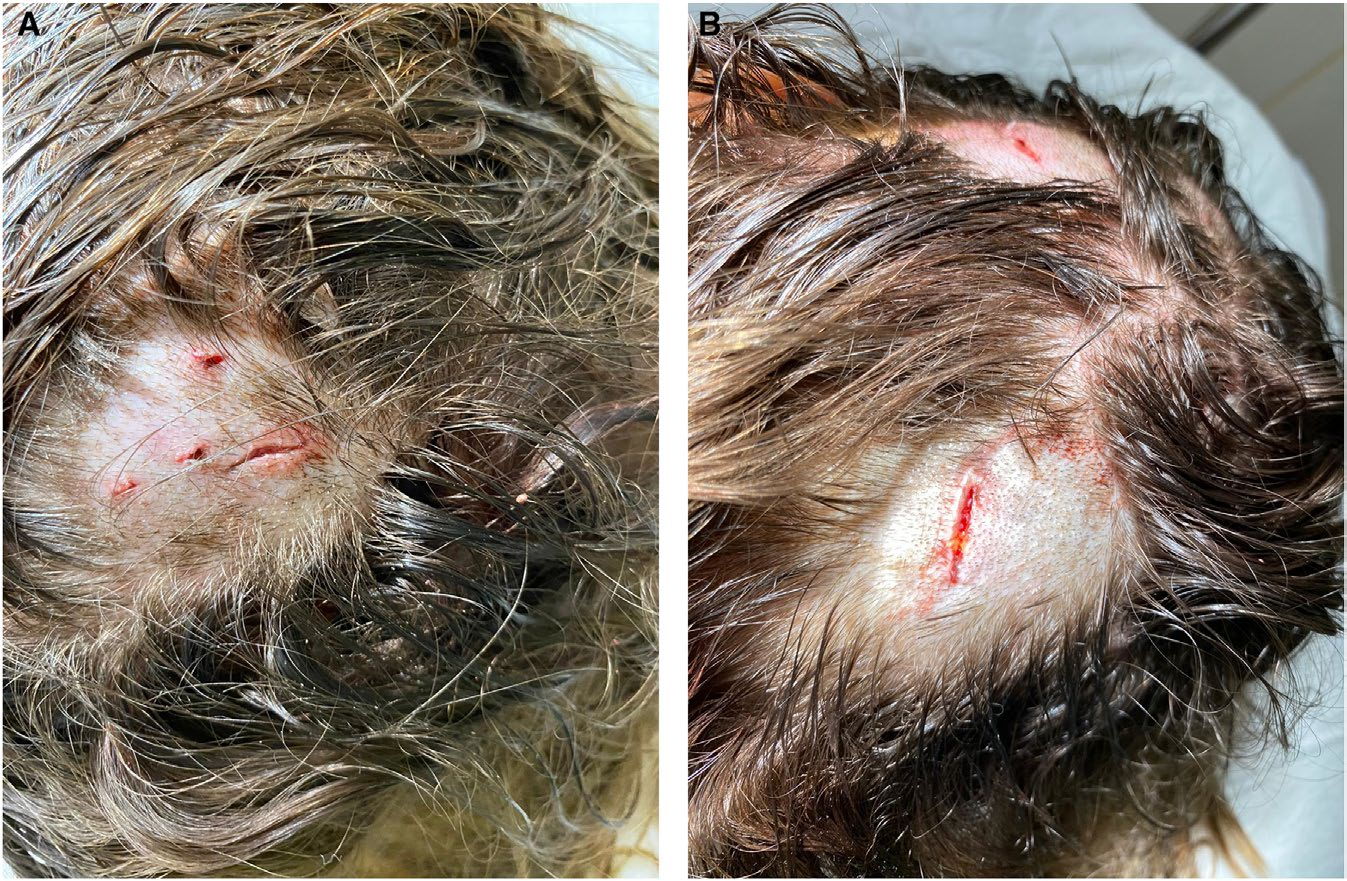
(A and B): Photos of patient's scalp wounds.

In October 2023, a group of 11 people, including the native guide, were walking along a footpath beside a small, forested river near a tourist camp in the rainforest in French Guiana. The camp is located 35 km from the nearest town Kourou in linear distance and requires around 1 h's drive and then 2 h and half by boat on the Kourou river to be reached. Most of the tourists were dressed in swimming trunks, as they were going to bathe in the river. A few minutes before the encounter with the Harpy Eagle, the group sighted a paca ( *Cuniculus paca* ) on the path, which surprised their guide as this animal is usually nocturnal. While the group was walking, a Harpy Eagle ( *Harpia harpyja* ) perched on a horizontal branch about 6 m above the group of tourists and began to observe them (Figure 1A,B). The guide said that a raptor of this species had already been seen several times on this path in recent weeks. In addition, a dead primate, possibly killed by this Harpy Eagle, had been found in the same area a few days earlier—a fact that was unknown to the tourist group by this point. The group was noisy, chatting and laughing while observing the bird, which seemed curious, deducing from the expanded facial disc directed into people sound sources. One of the people in the group, a 29‐year‐old woman, slightly built, measuring 170 cm in height and weighing 52 kg, walked back and forth along the path to make the bird change position so she could take better photos. After a long stop, the group gradually resumed their journey leaving the woman and her partner alone, after which they also started to leave. The bird then suddenly took off and swooped down on the young woman, who turned around and curled up in a ball, her head near her lap and her hands over her face. The bird of prey gripped at the back of her skull. The woman screamed in pain, and then fell silent, while the bird remained clinging to her head, albeit unstable, keeping its wings spread, its body leaning forward, its beak close to the ground. Her partner then tried to free the bird with his hands to no avail. He then slammed the eagle's head to the ground with his shoe, pressing down on its neck to immobilize it. The bird then suddenly released its hold, allowing the young woman to free herself and run away. The Harpy Eagle immediately took off again, seeming uninjured. It was seen a few minutes later and heard calling for quite a long time, although no vocalization had been heard either before or during the attack. No further attacks were reported in the following months.

**FIGURE 2:**
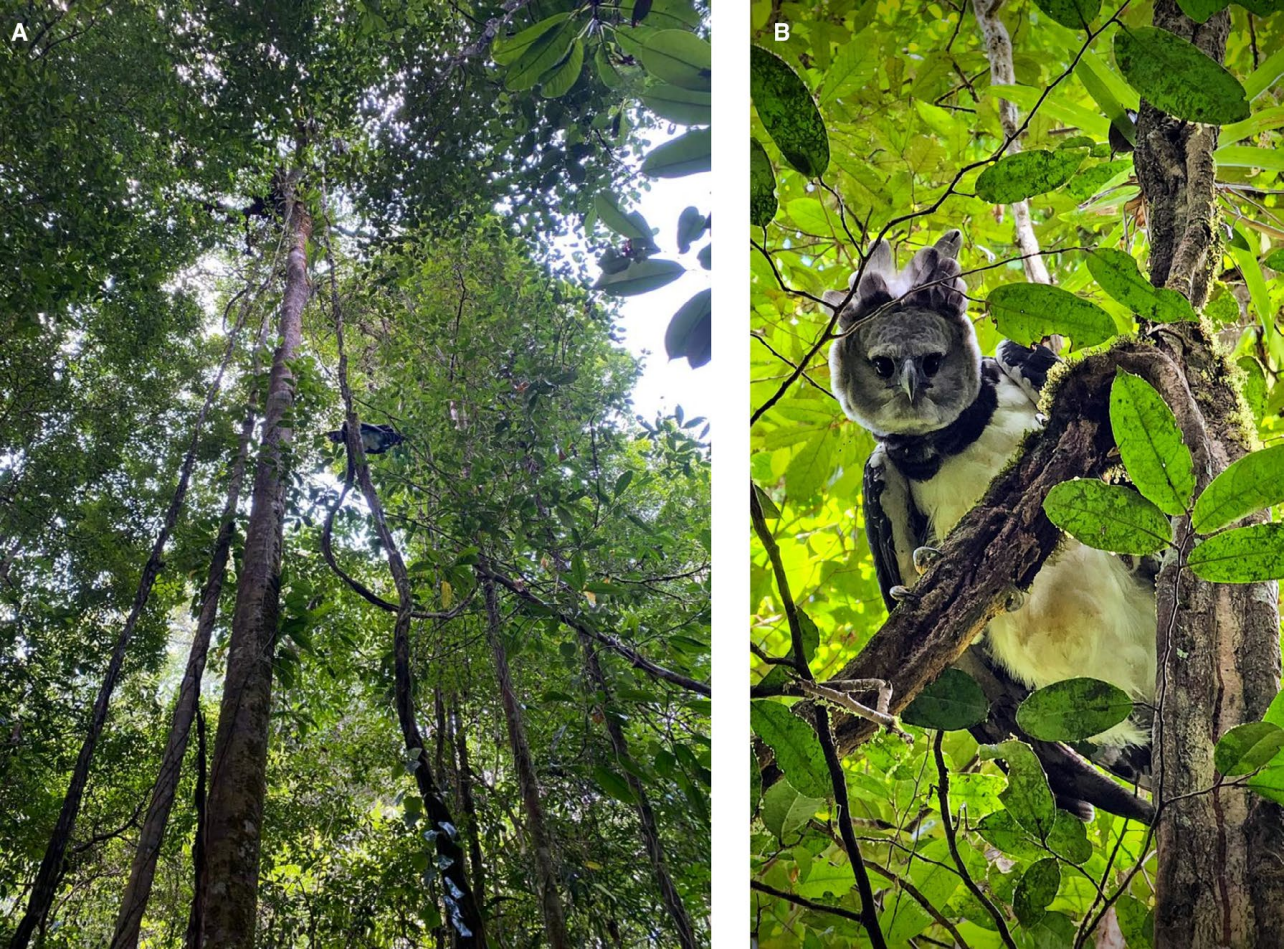
(A and B): Photos of the animal that attacked, (A) Showing the bird's distance from the ground, (B) Close‐up detail of the bird.

The young woman was taken to the coast 7 h later and to the emergency department of the Kourou Hospital. Three scalp wounds about 2 cm long were noted on the parietal, left parieto‐occipital, and right parieto‐occipital areas (Figure 2A,B). The patient's tetanus vaccination status was checked, and she was put on a seven‐day treatment of amoxicillin/clavulanic acid antibiotics. The skull wounds slowly improved over the following weeks.

## 4 Discussion

We present here the evidence indicating a new interaction between large raptors and humans, suggesting a reevaluation of the established framework within primatology and human evolution studies. Large eagles have demonstrated repeated instances of remarkable predation of large primates, notably navigating their antipredatory traits, wherein—as in the case presented here—sociality appears to play a significant role.

As apex predators of tropical and temperate ecosystems, eagles exhibit the capacity to occasionally subdue larger vertebrates, including large‐sized deer and antelopes, suggesting that the average prey size of certain giant eagle species—as well as the maximum carrying capacity on flight—might hinder their actual largest potential prey (Kerley and Slaght 2013). It is easy to suggest a fatal scenario if the Harpy Eagles mentioned here had attacked: (a) lethal body parts such as neck vertebrae, neurovisual or cardiovascular systems—as shown for other primates (Berger and McGraw 2007; Garbino et al. 2023; Thomsett 2015); (b) any body parts of human infants. However, most giant‐sized raptors are extinct, declining, or extirpated across significant portions of their habitats, as are many larger primates. Both factors negatively affect the opportunities to record such exceptional events. Through a review of the literature shown in the next paragraphs, we hope to challenge the prevailing view that raptors exert minimal predation pressure on anthropoids, potentially reshaping research frameworks in this domain.

Harpy Eagle attacks on humans are very rarely reported. Undocumented reports abound among Harpy Eagle researchers, coming from their own nest management activities or from tell‐tale stories from local communities. Recently in French Guiana, an attempted attack by this bird on a boat paddler was reported from the Kaw marshes, but the paddler avoided the attack and the eagle fell into the water (M. Cobigo, personal observation). Attacks on professionals to climbing up to the eagles nest for example to install camera traps are a common place, however more vicious when younger eaglets are in the nest (Parker 1999). This type of attack is relatively trivial and was recorded during the shooting of the BBC documentary “The Monkey‐Eating Eagle of the Orinoco” (Seymour et al. 2010). Individuals typically swooped into people during management activities of captive‐raised individuals reintroduced in Panamá, from which a few documented attacks are known. Attacks toward both people and other objects used in the management activities, such as quadbikes and motorcycle sits (Watson et al. 2016); Miranda personal observation. An individual may attack prey that is too large, as happens from time to time with the Golden Eagle on Alpine chamois ( *Rupicapra rupicapra* ) or the Spanish Eagle ( *Aquila adalberti* ) on roe deer ( *Capreolus capreolus* ). The African Crowned Eagle is known for its ability of regularly killing mammals up to 20 kg (Kemp 1994). Larger prey, however, cannot be carried away whole (McGraw et al. 2006), and African Crowned Eagles are unique on their capability of dismembering prey and caching parts of it.

We found only three cases of attack by a bird of prey in the medical literature, added to two literature reviews, summarizing the known attacks of raptors on humans (Olsen and Olsen 1980; Parker 1999). The author states that the subject is little covered in the scientific literature but is abundantly discussed in the local and regional press, as well as on the Internet. The first case involved a 41‐year‐old woman attacked by a Common Buzzard ( *Buteo buteo* ) while jogging in Switzerland (Ehrensperger et al. 2018). This was almost certainly a case of nest defense or impregnation. This attack resulted in tularemia. The second case involved an 80‐year‐old patient attacked on the hand by a Harris's Buzzard in Pakistan (Khan et al. 2011). It was most likely a falconry bird, and therefore imprinted, since this bird of prey is South American and not found naturally in Pakistan. These lesions were later complicated by cellulitis of the hand. No specific bacteria were found. Finally, a 51‐year‐old woman was attacked by a Golden Eagle ( *Aquila chrysaetos* ) in a cage (Muller and Kohnen 2005). She suffered severe damage to her eyeball. In French Guiana, nesting Roadside Hawks ( *Rupornis magnirostris* ), a common and relatively small bird of prey found in the Americas, regularly attack people in towns, particularly in suburban environments (unpublished records). In the present case, the hypothesis of a previously imprinted bird is implausible in the absence of any knowledge of a bird recently released in the vicinity of the attack site in particular, and more broadly, in French Guiana.

The idea of a bird defending the nest has also been suggested, as repeated sightings of the eagle in the same trail during consecutive weeks. However, no nest was detected in the area. In the case of the Harpy Eagle species, the nest is always in the canopy in the highest trees (Miranda et al. 2000). Although this suggests a potential defense context, the hypothesis is not plausible because Harpy Eagle tourism often involves the installation of towers near nests, as seen in Venezuela, Colombia, and the southern Amazon in Brazil, demonstrating that these birds tolerate human presence near their nests (Miranda et al. 2022). Another hypothesis is that the group interrupted a hunt, as suggested by the paca presence, or that the bird was defending or stressed out in regard to the primate it killed previously. One last hypothesis is that this was a particularly aggressive individual, which cannot be used to infer the average behavior of the species toward humans. Given the tolerance of Harpy Eagles to human presence near their nests, as seen in various ecotourism settings, and the impossibility of checking the presence of a nest in the area, it is more likely that the observed behavior was an isolated incident driven by situational stressors rather than a generalizable defensive or predation response of the species toward humans.

Many Harpy Eagle researchers are aware of other well‐documented cases of Harpy Eagle attacks on humans, often linked to the presence of prey on the ground, but they choose not to publish these incidents. These researchers fear that by publicly describing such attacks, they may inadvertently fuel laypeople's fear and subsequent persecution of these threatened raptors. Given that scientific papers are seldom read by the general public and are unlikely to reach wider audiences, we have decided to report this particular attack, hoping it will not negatively impact the perception of Harpy Eagles. Our aim is to contribute to scientific knowledge without fostering unwarranted fear or harm toward these eagles.

Sociality plays a crucial role in the defense mechanisms of primates, particularly in the context of predatory attacks by apex predators such as large raptors. The case described, along with numerous informal reports, highlights the increased likelihood of survival when individuals are not alone during such encounters. Had the individual in this incident been isolated, the outcomes could have been fatal, emphasizing the protective function of group dynamics in deterring or mitigating lethal attacks in both contemporary and evolutionary contexts.

## 5 Conclusion

Reports of large birds of prey attacking humans are exceedingly rare, but the case documented here of a Harpy Eagle attack in the Amazon rainforest highlights an unexpected predator–prey interaction with significant implications. Although the reasons for this attack remain speculative, the evidence suggests that large raptors like the Harpy Eagle may pose occasional but serious threats to humans under certain circumstances. This case, along with the prehistoric example of the Taung Child and the existence of now‐extinct giant raptors, reinforces the idea that avian predators likely played a more significant role in the evolutionary pressures faced by early hominins than previously thought. These interactions underscore the need for further research into the factors driving such encounters, while also emphasizing the importance of sociality as a critical antipredatory strategy in both contemporary and evolutionary contexts for primates, including humans.

## Author Contributions

**Loïc Epelboin:** conceptualization (lead), data curation (lead), formal analysis (lead), supervision (lead), writing – original draft (lead). **Rémi Mutricy:** writing – review and editing (equal). **Vincent Pelletier:** data curation (equal). **Alexis Fremery:** writing – review and editing (equal). **Maxime Dechelle:** writing – original draft (equal). **Otte Ottema:** supervision (equal), validation (equal). **Sébastien Pfefer:** writing – review and editing (equal). **Jenn Sinasac:** validation (equal). **Sylvain Uriot:** data curation (equal). **Oliver Claessens:** data curation (equal), validation (equal), writing – original draft (equal). **Everton B. P. Miranda:** supervision (equal), writing – original draft (equal).

## Conflicts of Interest

The authors declare no conflicts of interest.

## Data Availability

There is no data as such: this is an observation of a harpy attack in the forest. The article is based on the account of this attack, and all the information is provided in the manuscript.
